# If It Is Complex, Let It Be Complex – Dealing With Institutional Complexity in Hospitals: Comment on "Dual Agency in Hospitals: What Strategies Do Managers and Physicians Apply to Reconcile Dilemmas Between Clinical and Economic Considerations?"

**DOI:** 10.34172/ijhpm.2022.6922

**Published:** 2022-03-09

**Authors:** Thomas Andersson

**Affiliations:** ^1^School of Business, University of Skövde, Skövde, Sweden.; ^2^Faculty of Theology, Diaconal & Leadership, VID Specialized University, Oslo, Norway.

**Keywords:** Complexity, Institutional Logics, Hospital, Manager, Professional, Balancing

## Abstract

Waitzberg and colleagues identified strategies that managers and physicians in hospitals apply to reconcile dilemmas between clinical and economic considerations. Contributions that actually acknowledge the institutional complexity of hospitals and describe how to deal with it are rare. This comment explains the reason behind the institutional complexity in healthcare organizations and argues that institutional complexity is a good foundation for a well-functioning and sustainable healthcare, as long as we are able to deal with this complexity. This point underscores the importance of their contribution. However, even if the identified strategies on how to reconcile and balance different, competing demands are important, they are not easy to apply in practice. First, the strategies require frequent and high-quality interaction between different actors adhering to different institutional logics. Second, even when the strategies are applied successfully, it is difficult to make them sustainable since they rest on a fragile balance between competing logics. However, these are important avenues for future research for researchers who want to follow the route of Waitzberg and colleagues.

## Background

 Today’s hospitals work under a lot of pressure. On the positive side, research has made progress and made it possible to treat more diseases and save more lives. On the negative side, available resources do not increase at the same pace. This is an ever-lasting dilemma and it makes hospital care complex: there will always be many different, and often competing, demands and perspectives. Instead of pretending that there is a simple way forward, we should acknowledge this complexity, and instead focus on how people on the ground deal with this complexity in a positive way. Here, Waitzberg and colleagues made an important contribution by acknowledging the dilemmas of clinical and economic considerations in hospitals, as well as the dilemma of high-quality care vs. cost-efficient care, and focusing on constructive strategies that managers and physicians apply to reconcile and balance such dilemmas.^1^ This comment starts by elaborating and justifying the reason why I applaud Waitzberg and colleagues’ decision to let it be complex when it is complex. I then elaborate on why it might be even more complex than they suggest. Finally, I will discuss why although the identified strategies are important contributions, they are far from easy to apply in practice.

## Why There Is a (Good) Reason for Institutional Complexity in Healthcare Organizations

 The complexity in hospitals that Waitzberg and colleagues related to multiple objectives^1^ has been frequently described in research. Already 20 years ago, hospitals were pictured as consisting of different worlds, with different orders.^2^ However, these differences in culture, values and ways of looking at key issues were not seen as the main problem, since hospitals as organizations need to be able to meet many different – and sometimes competing – demands. The differences also encompassed different views of accountability/autonomy, systematization of clinical work, teamwork and power differentials, which made the different worlds were poorly integrated. This lack of integration was the main problem. The worlds tended to be held separately by different actors that preferred one of the worlds and thought the other worlds were problematic.^2^

 Currently, the institutional logics framework, which Waitzberg and colleagues relate to in their discussion,^1^ is often used to explain the complexity of hospitals. Because institutional logics capture that, it is a way of thinking that goes beyond the organization. Institutional logics guide social actions by providing assumptions and values on ways to interpret organizational reality.^3^ Thus, different institutional logics provide different meaning and values, and different interpretations of reality, which mean that they will give different steering signals on what to do in a situation. Institutional complexity means that many different institutional logics co-exist without any of them being able to dominate.^4^ This is an inevitable part of managing hospitals because we need all of these perspective and logics simultaneously if we are to have a sustainable well-functioning healthcare. All logics are “good at” prioritizing some important aspects, but none of them alone can ensure a well-functioning healthcare organization over time. The institutional complexity is actually inevitable for a well-functioning healthcare, but we need to be able to deal with this complexity.^5^

## Why Complexity Has Increased in Healthcare Organizations

 But why is not the institutional complexity just as high in any large organization? A business organization with thousands of employees should inevitably mean different co-existing and competing institutional logics, so why is such an organization less complex than a hospital? There are different competing logics in any organization, but in a business organization it is more likely that one logic is dominant, and most often the business logic. What causes the high complexity in public organization, and especially hospitals, is that the different logics are relatively equal in strength. No logic can dominate. As mentioned previously, this is an important prerequisite for a sustainable well-functioning healthcare organization over time, but it is also what causes the high institutional complexity^5^ and the need to balance different perspectives^6^ (logics) in every single situation.

 However, hospitals have not always been this complex to govern. In the 1960s and 1970s, hospitals were less complex because one logic was totally dominant: the medical logic of the physician profession. Since then, there has been a major growth of managerialism in hospitals.^7^ In countries where New Public Management (NPM)^8^ has been influential, this growth of managerialism has been represented by NPM. Today, there is an immense criticism of managerialism and NPM in hospital management in many countries, but perhaps this criticism should better acknowledge the unsustainable conditions of healthcare organizations before managerialism and NPM, when a professional logic ruled the game. The professional logic is “good at” quality and development, but its weakness is its lack of resource restrictions; it is always possible to do more and do better. When the whole healthcare sector was governed by this ideal, it gave an enormous expansion,^9^ which implied problems financing healthcare if the growth continued. Hospitals obviously also needed a resource perspective. This became one reason of the growth of managerialism and the beginning of what research later came to call NPM: public organizations imitated private business in terms of how to organize and manage their organizations. Managerialism and NPM made the management logic stronger, and thus increased the institutional complexity of hospitals because the new logic did not exchange existing logics, but was added to them.^10^ The paradox is that managerialism that should make hospitals more governable instead increased their complexity.^11^ This is currently the state of healthcare organizations: the institutional complexity is high. And it should be. Because if managerialism alone would be the ruling logic, it would probably not give a healthcare system that citizens would appreciate. On the other hand, if the professional logic alone would be the ruling logic, healthcare would have challenges to finance its activities. So, it if is complex, and should be complex, why is not everyone satisfied?

## Why We Pretend It Is Not Complex Instead of Acknowledging Complexity

 The main problem with complexity is that people prefer predictability over complexity.^12^ This is why people prefer to believe in the ability of different management concept to solve all problems in healthcare organizations.^13^ This is also why hospitals become fragmented as systems. Fragmentation is a common way to deal with complexity (or, more accurately, turn a blind eye towards complexity), because if the complex system is reduced to only one logic instead of several competing ones, the system appears to be more predictable. The problem is that since it is complex, actions based in only one logic will have a lot of unintended consequences.^14^

 There have been many studies on how managers and physicians fail to acknowledge the institutional complexity of healthcare, but Waitzberg and colleagues’ contribution instead focused on the perhaps rare occasion when they do succeed in reconciling and balancing competing logics and what strategies they use in these situations. This is important since it paves a way for how to better deal with institutional complexity in healthcare. Waitzberg et al^1^ (p. 8) identified three different strategies: “(1) reconciliation between economic and clinical considerations through increasing efficiency, which is possible only in those situations when there is no inherent conflict between these objectives. This is the case when activity-based payment incentivizes proper treatment; (2) the mitigation of dilemmas by reshaping managerial practices, such as treatment paths and coding; and (3) balancing considerations through reframing the focus of decision-making to bigger units of analysis.” These strategies constitute an important contribution, but I would consider an even more general common ground or condition of all of these strategies: the need for frequent and high-quality interaction between different actors. The first strategy acknowledges that even if two logics are in conflict, they will not be conflicting in all situations. Identifying situations where they do not conflict is the most obvious first step, but this requires interaction between different actors that often adhere mainly to one of the logics.^5,11^ Consequently, in practice, interaction is a precondition for reconciling the logics in this way. The other two strategies require change, either in managerial practices or focus, neither of which would be possible without interaction. Both require in-depth understanding of both managerial practices and clinical work, which is rare for a single actor to possess and usually requires interaction between different actors.

 Waitzberg and colleagues also touched upon why this is perhaps easier said than done, by describing the fragile balance between opposing forces or logics. Even when people have acknowledged the complexity, the preference for predictability does not disappear. A strategy that was fruitful in balancing opposing logics can destroy the balance if taken too far. Consequently, future research could continue on the route set out by Waitzberg et al by (1) investigating how the interaction that condition their identified strategies could be supported by creating arenas and roles that support such interaction^15^ and (2) making successful balancing sustainable; for example, by creating organizational structures that support balance between different interest and perspectives.^6^ If hospital organizations are complex, we should let them be complex and instead learn to deal with this complexity, similar to what Waitzberg et al did.

## Ethical issues

 Not applicable.

## Competing interests

 Author declares that he has no competing interests.

## Author’s contribution

 TA is the single author of the paper.
